# Early shell field morphogenesis of a patellogastropod mollusk predominantly relies on cell movement and F-actin dynamics

**DOI:** 10.1186/s12861-020-00223-3

**Published:** 2020-08-19

**Authors:** Weihong Yang, Pin Huan, Baozhong Liu

**Affiliations:** 1 Key Laboratory of Experimental Marine Biology, Center for Ocean Mega-Science, Institute of Oceanology, Chinese Academy of Sciences, 7 Nanhai Road, Qingdao, 266071 China; 2 Laboratory for Marine Biology and Biotechnology, Qingdao National Laboratory for Marine Science and Technology, Qingdao, 266000 China; 3 University of Chinese Academy of Sciences, Beijing, 100039 China

**Keywords:** Cell movement, F-actin dynamics, Mollusk, Morphogenesis, Shell field, Shell-formation gene

## Abstract

**Background:**

The morphogenesis of the shell field is an essential step of molluscan shell formation, which exhibits both conserved features and interlineage variations. As one major gastropod lineage, the patellogastropods show different characters in its shell field morphogenesis compared to other gastropods (e.g., the pulmonate gastropod *Lymnaea stagnalis*), likely related to its epibolic gastrulation. The investigation on the shell field morphogenesis of patellogastropods would be useful to reveal the lineage-specific characters in the process and explore the deep conservation among different molluscan lineages.

**Results:**

We investigated the early shell field morphogenesis in the patellogastropod *Lottia goshimai* using multiple techniques. Electron microscopy revealed distinct morphological characters for the central and peripheral cells of the characteristic rosette-like shell field. Gene expression analysis and F-actin staining suggested that the shell field morphogenesis in this species predominantly relied on cell movement and F-actin dynamics, while BrdU assay revealed that cell proliferation contributed little to the process. We found constant contacts between ectodermal and meso/endodermal tissues during the early stages of shell field morphogenesis, which did not support the induction of shell field by endodermal tissues in general, but a potential stage-specific induction was indicated.

**Conclusions:**

Our results emphasize the roles of cell movement and F-actin dynamics during the morphogenesis of the shell field in *Lo. goshimai*, and suggest potential regulators such as diffusible factors and F-actin modulators. These findings reflect the differences in shell field morphogenesis of different gastropods, and add to the knowledge of molluscan larval shell formation.

## Background

As a key characteristic in the phylum Mollusca, the shells protect the soft body parts and have contributed to the persistence of this animal lineage since at least the early Cambrian period [1, 2]. The tissues responsible for shell formation start their specification at early developmental stages (e.g., the gastrula stage) and experience complex changes with development [3–7]. Here, we refer to these tissues as the “shell field”, although this term was used to specifically indicate the tissues at a relatively late stage (i.e., the stage after evagination) [7]. The development of the shell field represents the period during which the tissues obtain the capacity to secret larval shells, and thus is essential to understand the mechanism of shell formation.

Although its origin can be traced back to early embryonic stages (as early as the 16-cell stage) [7–9], the shell field is the first morphologically discriminable shell-formation tissue during ontogenesis. It is typically organized as a regular central-to-peripheral rosette pattern, in which the central and peripheral cells show distinct characteristics (at least for conchiferan mollusks, which comprise of most of the extant molluscan species) [3–7, 10, 11]. Based on classical microscopic observations, previous studies reveal many important aspects of shell field morphogenesis, which provide a general outline of the process (e.g., the thickening of dorsal tissue as a common initial step) and reveal interlineage variations among different mollusks [7].

In recent years, more techniques such as the confocal laser scanning microscopy (CLSM) and cell-lineage tracing are employed and they have provided additional details of shell field development [9, 12–14] (although shell formation is not the primary focus for some of the studies). Gene expression is another useful tool to study early shell formation, which can be used to mark the morphologically similar cells in the shell field based on given types of mRNA. Marking cell populations using gene expression data is particularly applicable given that an increasing number of genes showing expression associated with larval shell formation (termed the potential shell formation genes, pSF genes) are identified in the past two decades [15–19].

Using the multiple techniques mentioned above, researchers have re-investigated the shell field morphogenesis in the pulmonate gastropod *Lymnaea stagnalis*, which has been a model system to study molluscan shell formation [12]. The results reveal undescribed details of the process, such as the establishment of contact between ectodermal and endodermal cells and the dynamics of endogenous peroxidase and alkaline phosphatase activities inside the shell field [12]. These findings provide fundamental supports for understanding larval shell formation. In particular, they support the existence of an ancestral process that the formation of molluscan shell field is induced by endodermal tissues [12]. On the other hand, lineage-specific characters are frequently observed in the shell formation of different mollusks [5, 7, 10–12, 20, 21]. Other mollusks could show very different characters in shell field morphogenesis that may be associated with varied developmental strategies (e.g., the manners of gastrulation). For instance, in the major gastropod clade Patellogastropoda, many species (such as *Patella vulgata*) showed mainly epibolic gastrulation (in which the micromere-derived epidermal tissues move and expand to internalize the macromeres), different from the gastrulation of *Ly. stagnalis* involving mainly invagination [22]. Previous studies suggested that in mollusks with epibolic gastrulation, the shell field morphogenesis may not result from the inductive roles of the endoderm since the endoderm-ectoderm contacts are not interrupted [7]. However, this notion requires further certification [12].

In the present study, we analyzed the shell field morphogenesis in the patellogastropod *Lottia goshimai* using electron microscopy, CLMS and pSF gene expression analysis. Our results reveal predominant roles of cell movement and F-actin dynamics during the process. Inductive roles from endodermal tissues are not supported in general, while such effects confined to late developmental stages were suggested.

## Results

### Morphological changes during shell field formation

**Fig. 1:**
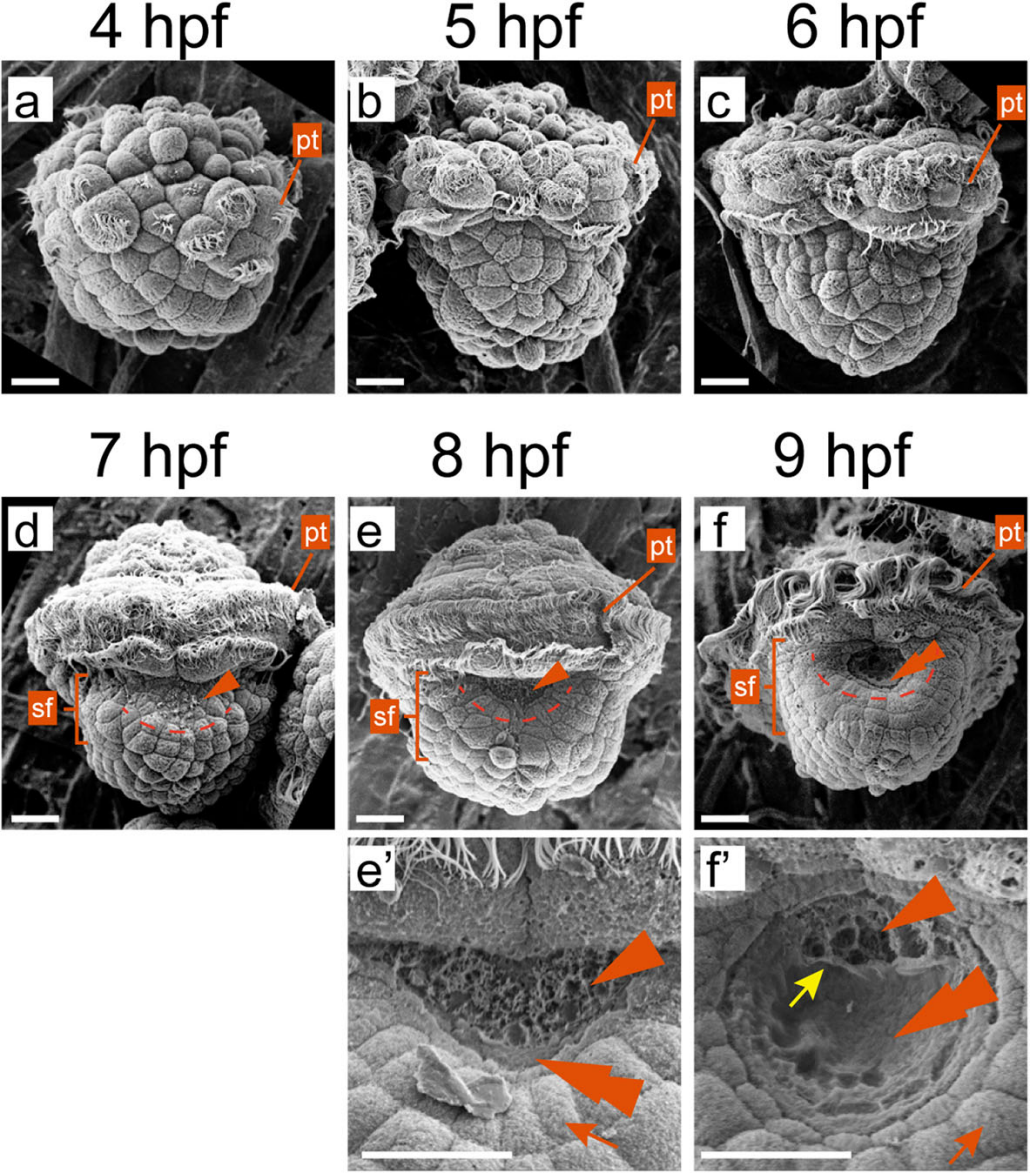
SEM images showing the formation of the shell field in *Lo. goshimai*. All panels are dorsal views with anterior to the top (except **a**, since the dorsal side could not be readily determined at the developmental stage). Panels **e**’-**f**’ are images with higher magnifications showing the details of the shell field; note that they were obtained from different individuals from those shown in **e** and **f**. A shell field (sf) is discriminable with the development of some cells with surface elaborations at 7 hpf (the arrowhead in **d**). The peripheral cells started to arrange in an imaginary circle since 7 hpf (dashed curves in **d**-**f**) and exhibited obvious wedge-shapes since 8 hpf (highlighted by the orange arrows in **e’** and **f’**). The shell formation started at around 8 hpf and a shell plate is evident at 9 hpf (double arrowheads in **e’**, **f** and **f’**). The yellow arrow in **f’** indicate the apparent correlation between surface protrusions and the shell plate. Note that although we label the prototroch (pt) in all panels, it was not fully developed at 4 hpf. Bars represent 20 μm

Similar to other patellogastropods such as *P. vulgata* [23, 24], the first two rounds of cleavages were equal in *Lo. goshimai*, and the gastrulation started since the 64-cell stage at around 3–3.5 h post fertilization (hpf). At this stage, the mesodermal somatoblast cell (4d) formed, and the macromeres at the vegetal pole began to invaginate. The subsequent development involved extensive cell movements (mainly epiboly), and the embryos started to swim in the seawater at 4–5 hpf with the development of ciliated cells (trochoblasts, some of which will ultimately form the prototrochal ciliary band that allows the larva to swim) (Fig. 1a-b). A shell field was discriminable on the dorsal side of the embryo since 7 hpf (Fig. 1d). The newly-formed shell field at 7 hpf could be recognized based on the characteristic short protrusions on the surface of some dorsal cells (Fig. 1d). Although similar superficial protrusions in the shell field were determined to be microvilli or lamellipodia in some other mollusks [5, 10, 11, 20], we could not determine their nature based on our results (Fig. 1e’). In subsequent development, the cells with surface elaborations slightly invaginated (Fig. 1e). Other dorsal ectodermal cells surrounding them, which were irregularly arranged at earlier stages (Fig. 1b-c), started to arrange into an imaginary circle (Fig. 1d-f) and transited into wedge shapes (orange arrows in Fig. 1e’-f’). At 8 hpf, the shell field exhibited a typical (despite partially) rosette-like pattern and occupied most of the area of the dorsal ectoderm (Fig. 1e). At 9 hpf, the rosette-like shell field was well developed, and a shell plate formed in the central of this area (double arrowheads in Fig. 1f, f’). We found that the shell plate was frequently discontinuous along its anterior margin at 9 hpf (in 5 of 6 recorded individuals) and showed apparent associations with the surface protrusions in the central region (the yellow arrow in Fig. 1f’). However, the anterior edge of the discontinuous shell plates did not show uniform morphology between individuals and were more like a result of the rupture of an intact shell, suggesting the discontinuity of the shell plate (and the seemingly association with the surface protrusions) might be an artifact caused by technical reasons during sample preparation.

Notably, here and in the subsequent text we describe the body axes of the embryos based on the orientation of a pre-torsion embryo, which are comparable to those of most other animals and different from those of a post-torsion embryo. During ontogenetic torsion in gastropods, the shell and visceral mass (visceropallium) rotate by 180 degree with respect to the head and foot (cephalopodium) (it occurs at around 22–26 hpf in *Lo. goshimai*); and this process affects the orientation of the shell field. The anterior-posterior axis of the shell filed are inverted during torsion, which indicates that the posterior rim of the shell field mentioned here will comprise of the anterior margin of the larval mantle in the post-torsion larvae. At the same time, the “dorsal” side we described here will become the ventral side of the visceropallium after torsion.

### pSF gene expression indicates widespread cell movement during shell field morphogenesis

We analyzed the expression of several pSF genes aiming to trace the dynamics of potential different cell populations. Four genes were used, including BMP2/4, Engrailed, Hox1 and GATA2/3. These genes were considered to be pSF genes due to the association of their expression and larval shells [15–17, 19, 25, 26], and we confirmed their expression in the shell field of *Lo. goshimai* in our recent works [27] (Tan et al., in preparation).

**Fig. 2:**
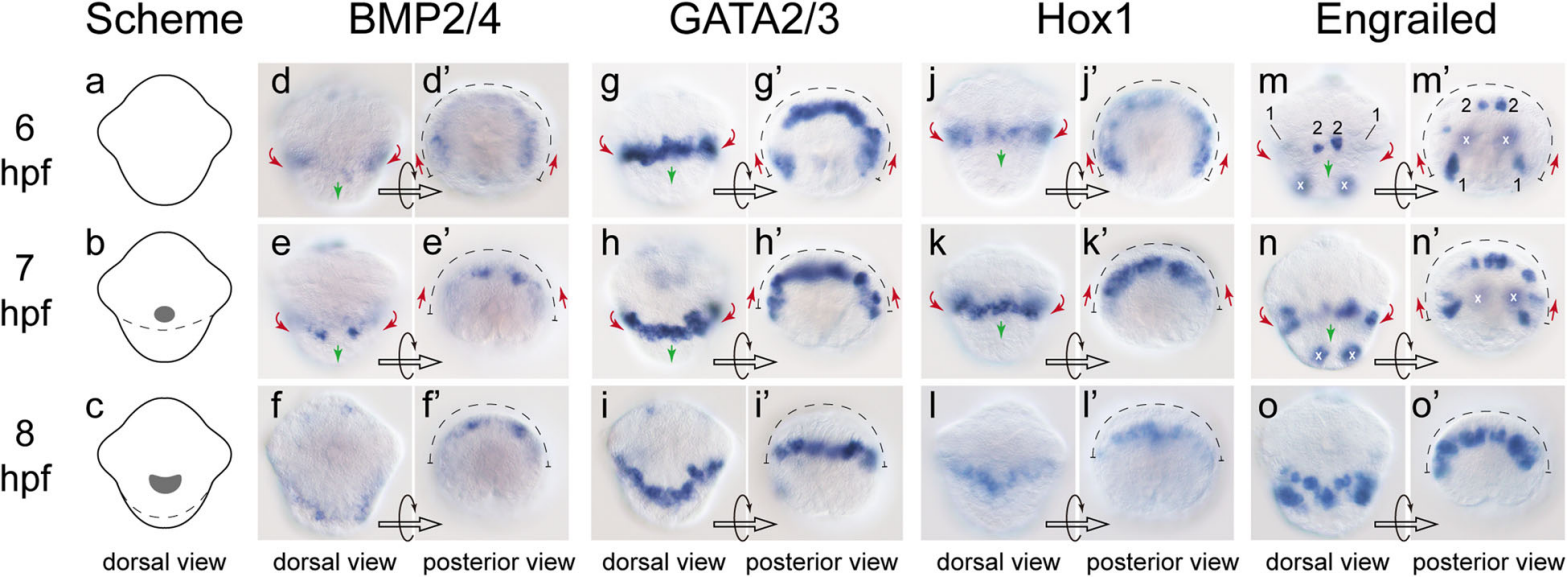
Expression of the four pSF genes from 6 to 8 hpf. Panels **a**-**c** are schematic diagrams showing the development of the shell field during this period (dorsal views). The shaded areas indicate the central region with surface elaborations (Fig. 1), and the dashed lines indicate the territory of the shell field roughly estimated based on the wedge-shaped peripheral cells (Fig. 1) and the expression of pSF genes shown in panels **d**-**o**. Panels **d**-**o** are dorsal views, anterior to the top; and panels **d’-o’** are posterior views, dorsal to the top. The pSF gene expression spanned relatively wide ranges at 6 hpf, which aggregated to the dorsal side in the subsequent development (indicated by red arrows in **d**-**o** and **d’**-**o’**). At the same time, their dorsal expression moved posteriorly (indicated by green arrows in **d**-**o**). The white crosses in **m**-**o** and **m’-o’** indicate the Engrailed expression in the cells that do not contribute to shell field development. For clarity, Engrailed expression domains at 6 hpf are indicated by numbers (1 and 2 in **m** and **m’)** given that the signals were distributed on dorsal and ventral sides and it was relatively difficult to discriminate them in different panels

The genes started their expression in posttrochal cells between 4 and 5 hpf, despite the pretrochal BMP2/4 expression at earlier stages (Supplemental fig. S1). In subsequent development, the expression of the genes changed continuously. Expression of BMP2/4, GATA2/3 and Hox1 transited into continuous patterns, while scattered Engrailed expression sustained in general (Fig. 2). We determined that all cells expressing BMP2/4, Hox1 and GATA2/3 contributed to shell field development (Fig. 2d-l). For Engrailed, although the gene also participated in other processes, we could determine which part of Engrailed expression contributed to shell field development by comparing the expression of sequential developmental stages (Fig. 2m-o).

We deduced that the changes in the pSF gene expression were caused by the movement of shell field cells. Such cell movement, which was evident from the posterior view (Fig. 2d’-o’), was reflected by the continuous changes in the locations of the most distant gene expression regions (with respect to the middle line of the dorsal side). Specifically, at 6 hpf, the most distal expression of each gene was distributed laterally (or even partially ventrally) (red arrows in Fig. 2d’, g’, j’, m’). In the subsequent development, these distal gene expression regions moved continuously toward the dorsal side (highlighted by the red arrows in Fig. 2d’-e’, g’-h’, j’-k’, m’-n’). At 8 hpf, most of the gene expression were detected on the dorsal side (Fig. 2f’, i’, l’, o’). In accordance, from the dorsal view, the gene expression patterns transited from straight lines to curved lines (indicated by green arrows in Fig. 2d-o), suggesting an expansion of the shell field with the migration of the cells from the ventral and lateral sides.

### F-actin dynamics: cell shape change, ectoderm-meso/endoderm contacts and a boundary between ectodermal tissues

**Fig. 3:**
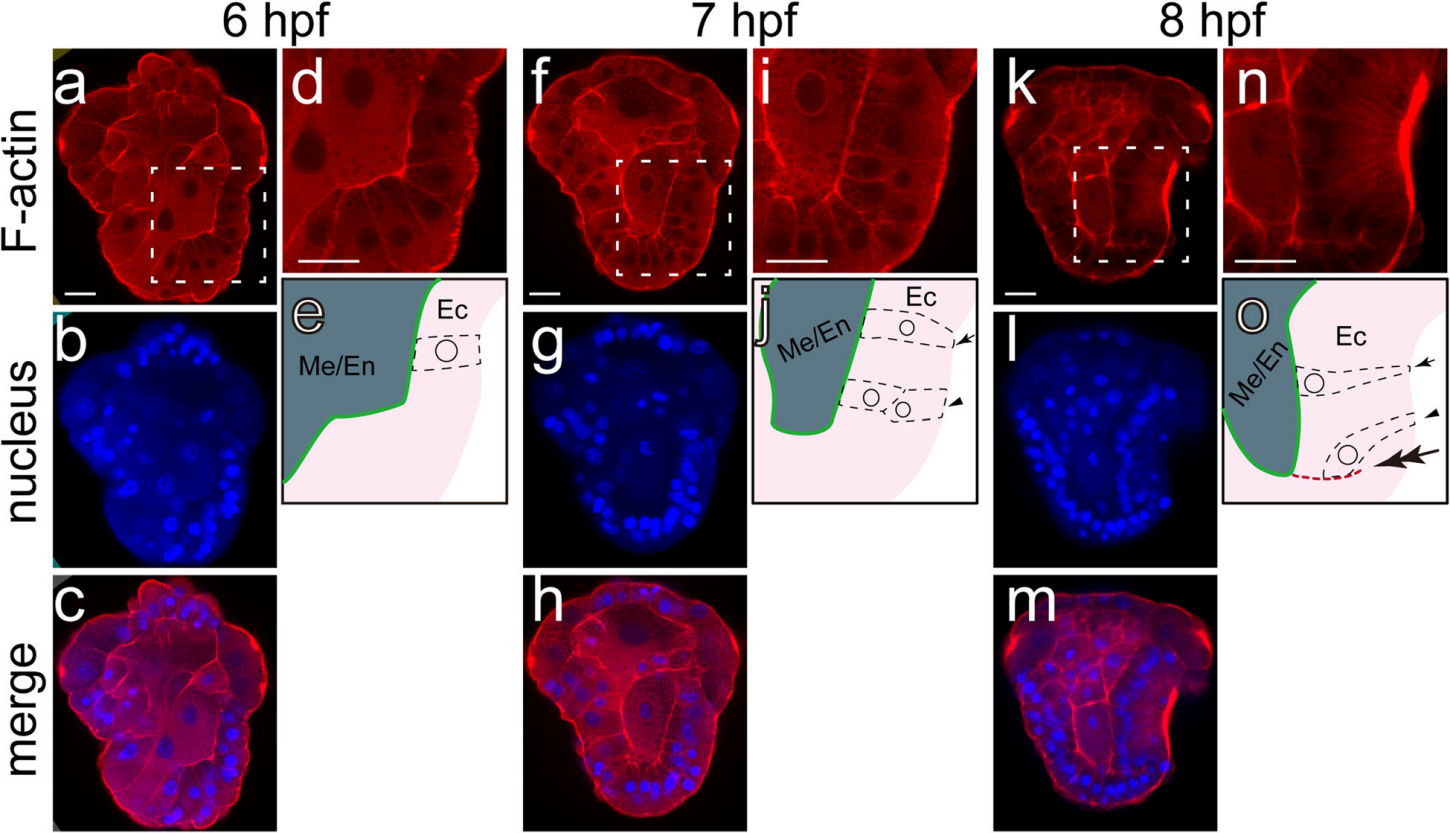
Phalloidin staining showing the dynamics of F-actin during shell field morphogenesis. Distributions of F-actin (phalloidin staining), nucleus (DAPI staining), and merged images at 6–8 hpf are shown in **a**-**c**, **f**-**h** and **k**-**m**, respectively. All panels are lateral views with dorsal to the right and anterior on the top (CLSM optical sections). **d**, **i** and **n** are magnified images of the enclosed regions in **a**, **f** and **k**, respectively; and **e**, **j** and **o** are corresponding schematic diagrams. In the diagrams, the contacts between ectodermal (Ec) and meso/endodermal (Me/En) tissues are indicated by green lines. In **j** and **o**, the typical shapes of a central cell (arrows) and a peripheral cells (arrowheads) in the shell field are shown. The red dashed line in **o** (highlighted by the double arrow) indicates the evident aggregation of F-actin that was located in the posterior part of the embryo and separated ectodermal cells

We investigated the dynamics of filamentous actin (F-actin) from 6 to 8 hpf using phalloidin staining, and revealed evident changes in the cell shape and cell-cell contacts and the formation of an F-actin-based boundary between epidermal tissues (Fig. 3). At 6 hpf, when the shell field was not morphologically recognizable, the dorsal ectodermal cells showed generally column-shapes (Fig. 3d, e). There were evident contacts between ectodermal and meso/endodermal tissues at this stage (Fig. 3d, e). At 7 and 8 hpf, when the shell field showed a central-to-peripheral rosette pattern (Fig. 1d, e), different F-actin distribution patterns were detected at the two developmental stages. This difference reflects F-actin dynamics during the period, itself distinct for the central and peripheral cells (indicated by arrows and arrowheads in Fig. 3j, o). For the central cells, they were elongated at 7 hpf and transited into flask shapes at 8 hpf (Fig. 3i, j, n, o). The elongation of these cells caused the thickening of the tissues and marked the formation of the shell field. The apical sides of these cells concentrated in the central region of the shell field at 8 hpf and showed strong phalloidin staining, coinciding with the distribution of the surface protrusions in this invaginated region (Fig. 3n, o). Given these flask-shaped cells had contracted apical sides, the strong phalloidin staining on the apical side may also suggest the distributions of contractile microfilaments in this region. Moreover, the contacts between these central cells and meso/endodermal cells were not interrupted during this period (indicated by green lines in Fig. 3e, j, o). For the peripheral cells, on contrast, they were not evidently elongated at 7 hpf. Alternatively, they were apparently organized into double layers, although we could not determine whether they were pseudostratified tissues (Fig. 3i, j). At 8 hpf, these cells also transited into flask shapes; and their apical sides were located in more peripheral regions, which seemed to be outside of the invaginated region (for at least a part of them) (Fig. 3n, o). Noticeably, the contacts between the peripheral cells and meso/endodermal cells were lost at this stage, and the peripheral cells only contacted with other ectodermal cells (Fig. 3n, o).

Another notable result is that an aggregation of F-actin was detected on the basal side of the peripheral cells of the shell field at 8 hpf (highlighted by the double arrowhead in Fig. 3o), indicating the existence of a developmental boundary between adjacent tissues.

### No obvious contribution of cell proliferation to early shell field morphogenesis

**Fig. 4:**
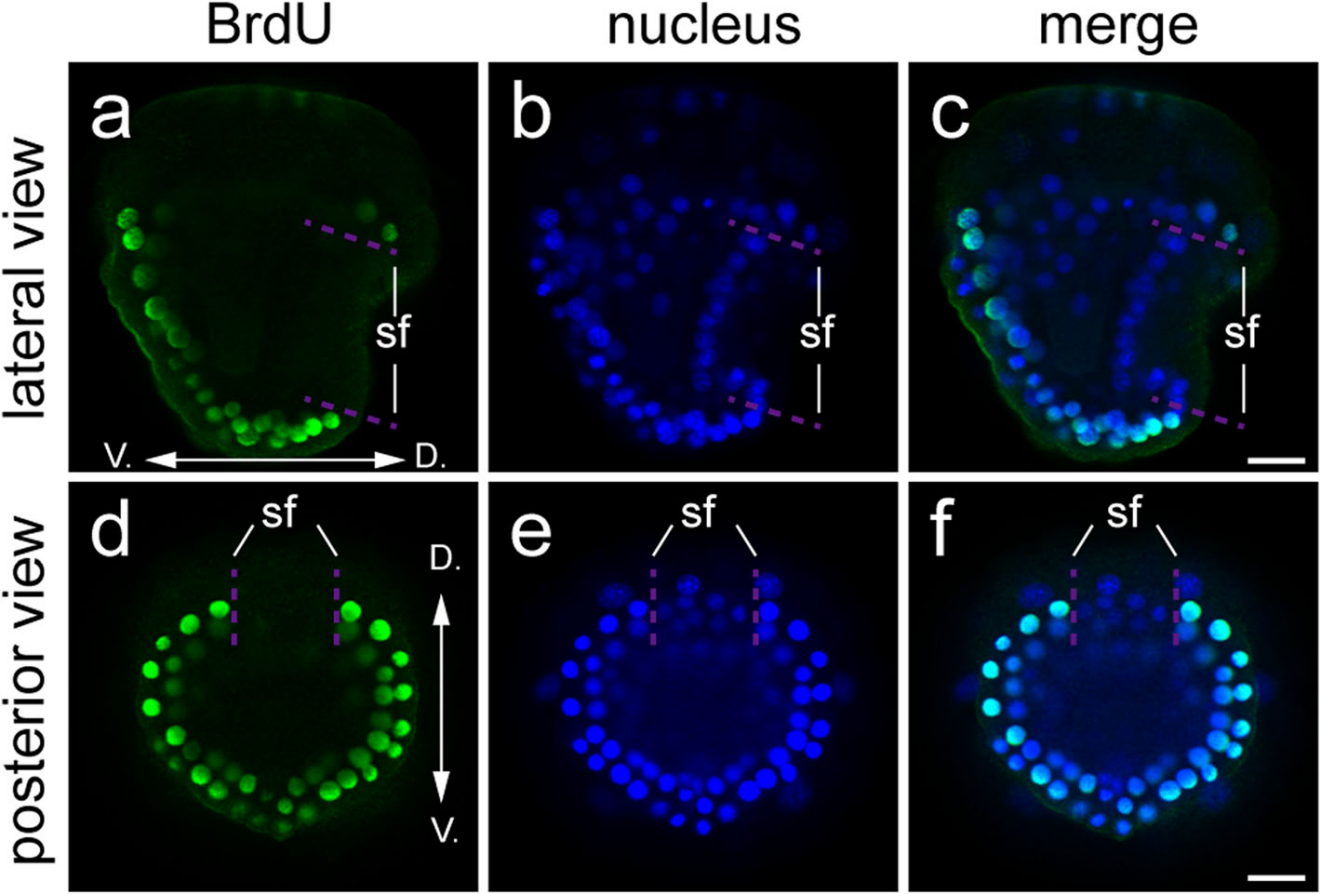
BrdU assay revealed no evident cell proliferation in the shell field from 7 to 8 hpf**.** All panels are CLSM optical sections. BrdU was added at 7 hpf and the samples were collected at 8 hpf. The incorporation of BrdU (green fluorescence) indicates the divided cells during this period. No evident cell proliferations was detected in the shell field (sf). Similar results were revealed for an extended period (6–8 hpf, see supplemental figure S2). D, dorsal; V, ventral. Bar represents 20 μm

The gene expression data, especially the changes in pSF gene expression from 7 to 8 hpf (Fig. 2d-o), suggest that the shell field expanded during this period. While cell movement and cell shape change should contribute to such expansion, cell proliferation may also be involved in the process. To test this speculation, we used a BrdU (5-bromo-2′-deoxyuridine) assay to evaluate the contribution of cell proliferation to the process. The nucleotide analog BrdU was added to the seawater at given time points, and the samples were collected after a determined period (1 or 2 hours). In this way, BrdU was incorporated into the nuclei of all cells that divided during this period and thus reflected the state of cell proliferation. Between 7 and 8 hpf, although we detected obvious cell proliferations in non-shell field ectodermal tissues (e.g., the ventral ectoderm), no cell proliferations was revealed in the shell field (Fig. 4). Further analysis focusing on an extended period (from 6 to 8 hpf) confirmed this finding by showing similar results (supplemental fig. S2). In the control group, the same amount of DMSO was added to the seawater and no BrdU antibody staining was detected as expected.

## Discussion

### Early shell field morphogenesis of *Lo. goshimai*: morphological characteristics

The morphogenesis of the shell field was quick in *Lo. goshimai*. A typical rosette-like shell field formed in approximately 2 h at 25 °C (from 6 to 8 hpf). SEM observations revealed two major morphological changes during this process. First, a central area with surface elaborations developed (since 7 hpf), which then invaginated and expanded in the subsequent development (Fig. 1d-f). The second change is that the peripheral cells of the shell field transited into wedge-shapes and were arranged in imaginary circles (Fig. 1d-f). Similar morphological characteristics, e.g., the surface elaborations in the central cells and the rosette-like organization, were also observed in the shell field of the gastropod *Ilyanassa obsoleta*, another model system used to study molluscan shell formation [10]. Similar protrusions on the surface of cells, which were determined to be microvilli, were observed in the shell field of the gastropod *Biomphalaria glabrata* [11]. In contrast, no obvious superficial protrusions is observed in the central region of the shell field of *Ly. stagnalis* embryo [12].

Previous studies revealed evidence that the initial shell plate was secreted by the peripheral cells of the shell field [7, 10–12, 28]. On contrast, we noticed an apparent association between the shell plate and the central cells at 9 hpf (Fig. 2f’). This result suggests a potential role of shell secretion of the central cells, which, however, was not reported before. Since the morphological characters of the discontinuous shell plate suggest it was derived from the rupture of an intact shell plate, we speculated such “association” might be an artifact caused by technical reasons during the SEM process. Further studies using an optimized SEM procedure are required to clarify this question.

### Implications for the mechanisms of larval shell formation

Previous studies on gastropod embryos generally focused on the dorsal ectodermal tissues, the majority of which indeed contributed to the subsequent shell field development [10, 12]. Somewhat unexpectedly, our results revealed that the lateral or even ventral tissues contributed to shell field development in *Lo. goshimai*. Despite this unexpectation, nevertheless, this finding actually shows consistency with some current knowledge. It explains why all 2q blastomeres contribute to the larval mantle of some patellogastropods (e.g., *P. vulgata* [8]). Based on the relationship between the body axis and the locations of early blastomeres (D (or 2D) to the dorsal) [29, 30], it is relatively difficult to explain the inclusion of 2b lineage in the larval mantle of the pre-torsion embryos (especially their relatively anterior distributions) [8], since the 2b-descedants are expected to be distributed on the ventral side. Our results suggest that some ventral cells, which may include 2b-descedants, migrate to the dorsal side and contribute to the shell field formation. Moreover, the involvement of such cell movement also provides insights into the mechanisms of shell field morphogenesis. The relatively wide range of cell movement indicate that this process may be coordinated by diffusible factors. In this context, the BMP signaling may be a candidate regulator, given that the signaling has been revealed to play essential roles in shell formation [16, 18, 31].

Several other aspects of shell field morphogenesis in *Lo. goshimai*, i.e., the development of surface protrusions in central cells, the cell shape changes throughout the process, and the F-actin-based boundary formed after 8 hpf, all possibly involve the regulation of F-actin. In previous reports, lamellipodia or microvilli (both are F-actin-related structures) were also frequently observed in the shell field of mollusks [5, 10, 11, 20, 32]. These observations suggest the functions of F-actin in molluscan shell formation, and it would be intriguing to explore the roles of F-actin regulators in the process (e.g., the Rho subfamily of small GTPases [33]). In addition, we revealed the existence of a boundary adjacent to the edge of the shell field (reflected by F-actin aggregation in this region; the double arrow in Fig. 3o). This result supports the involvement of a (F-actin-based) developmental boundary in the larval shell formation of mollusks that has been proposed long before [15]. This boundary may separate the shell field and other ectodermal tissues, or, it may be located inside the shell field, given that the precise determination of the territory of shell field would require assistance of other indicators.

An inductive role of the endoderm to the formation of the molluscan shell field has been proposed for a long time [7, 12]. In *Ly. stagnalis*, this viewpoint is strongly supported by the fact that the dorsal ectoderm only starts its invagination after the contact with the endodermal tissues is stably established [12]. However, it has been noted that in the species with epibolic gastrulation “the contact of the cells is never interrupted” [7]. Similar to *P. vulgata* [22, 23], the gastrulation of *Lo. goshimai* involved mainly epiboly. Our results confirmed that tight contacts between ectodermal and endo/mesodermal tissues were constant before the formation of a morphologically discriminable shell field and during the subsequent shell field development in *Lo. goshimai*. These results support that no inductive signals from the endodermal tissues exists in the shell field morphogenesis in the species. Nevertheless, as speculated before, despite the constant contact between ectodermal and meso/endodermal tissues, there is a possibility that the meso/endodermal tissues may only acquire the capacity to induce shell field formation at relatively late developmental stages [7]. This scenario is supported by our results that at the stage when evident invagination of the shell field occurred (8 hpf), the contacts with meso/endodermal tissues only sustained for those invaginated cells in the central region, while the peripheral, un-invaginated tissues apparently lost such contact (Fig. 3). In summary, our results indicate either no inductive roles from the meso/endodermal tissues to shell field morphogenesis or inductive roles confined to relatively late developmental stages.

The BrdU assay revealed little contribution of cell proliferation to early morphogenesis of the shell field in *Lo. goshimai*. Such lack of cell proliferation is somewhat unexpected given the evident expansion of the shell field during the period we investigated (Fig. 2d-o). This result is also very different from the previous reports that reveal active cell proliferation in the shell field [7]. We propose this difference may represent variations between different developmental stages. The developmental stages we addressed represent only the early stage of shell field development in *Lo. goshimai*. In subsequent development (i.e., after 10 hpf), the shell field experiences quick growth till at least 24 hpf when the larval shell can enclose the whole larval body (partially reported in our recent work [27]). Involvement of cell proliferation in the shell field development (if exists) may be detected in these late developmental stages. Alternatively, the different results regarding the contribution of cell proliferation in the shell field between our study and previous studies [7] may also reflect inter-lineage variations. In patellogastropods such as *Lo. goshimai*, the larval shell does not show evident growth in late larval stages (e.g., after 24 hpf) before metamorphosis (after 60 hpf). In contrast, the larval shell shows continuous growth before metamorphosis in some other gastropods (e.g., *Polinices pulchellus* [34]). It would be intriguing to explore whether the different states of cell proliferation in the shell field would be associated with the varied larval shell growth manners among different gastropod lineages.

We revealed that although the formation of the highly regular rosette-like shell field occurred in relatively late stages (7–9 hpf), the pSF gene expression was detectable much earlier (5–6 hpf). These results indicate that the specification of shell field tissues seemingly does not rely on a particular organization pattern of the shell field (the rosette-like pattern), and that the formation of a morphologically discriminable shell field and the specification of shell field cells may be decoupled. It is consistent with previous observations that in some circumstances when the formation of shell field is interrupted, the differentiation of shell field tissues is somewhat uninfluenced since birefringent shell materials can be frequently observed [35–42]. The particular organization of the shell field may be only necessary for the correct formation of a shell plate, but not for the specification of particular cell types. Nevertheless, since the roles of the pSF genes we investigated remain largely unknown, we do not deny the possibility that the early expression of these pSF genes actually does not mark the specification of shell field cells. Functional study on these genes are required to test whether the cell specification and shell field morphogenesis are decoupled and to elucidate the roles of the two processes in the formation of larval shell.

## Conclusions

We revealed several essential aspects during the early shell field morphogenesis of *Lo. goshimai* that seem to differ from some other gastropods. These differences reflect the specie variations during shell field morphogenesis, which could be related to the developmental strategies of different species and the resultant variations in the gastrulation (epiboly, invagination etc.) and larval types (whether a trochophore larva is developed). We emphasized the roles of cell movements and F-actin dynamics in shell field morphogenesis of *Lo. goshimai*. It would be intriguing to explore the roles of potential regulators, such as BMP signaling and Rho members. It is possible that although the shell field morphogenesis differs among species, they may share common regulators that are inherited from their common ancestor.

## Methods

### Animal and sample collection

Adult *Lo. goshimai* Nakayama, Sasaki & Nakano, 2017, were collected from intertidal rocks in Qingdao, China. According to the national regulation (Fisheries Law of the People’s Republic of China), no permission is required to collect the animals and no formal ethics approval is required for this study.

Spawning occurred after the animals were transferred to the laboratory. To collect the two type of gametes, each single individual of the animals was placed in a 100-mL plastic cup containing approximately 50 mL seawater. After spawning, artificial fertilization was performed by adding sperm to the oocyte suspension, and extra sperm was washed using filtered seawater (FSW). The fertilized eggs were cultured in FSW containing antibiotics (100 unit/mL benzylpenicillin and 200 μg/mL streptomycin sulfate) in 100-mL cups and were incubated at 25 °C in an incubator. Developmental stages were referred to hpf.

At desired developmental stages, the samples were collected using 200-mesh cloth and fixed in 4% paraformaldehyde (PFA) (1 × PBS, 100 mM EDTA, 0.1% Tween-20, pH 7.4) overnight at 4 °C. Then the samples were transferred to methanol and stored at − 20 °C (for whole mount in situ hybridization [WMISH]) or transferred to PBST (1× PBS, 0.1% Tween-20, pH 7.4) and stored at 4 °C (for phalloidin staining). For SEM, the samples were fixed in 2.5% glutaraldehyde (diluted in FSW) overnight at 4 °C, transferred to PBST and stored at 4 °C.

#### Genes

The four pSF genes we used in the present study (BMP2/4, GATA2/3, Engrailed and Hox1) were retrieved from a developmental transcriptome of *Lo. goshimai* (deposited in the NCBI SRA database, accession NO. SRX3353365) in our recent works [27] (Tan et al., in preparation).

#### SEM, WMISH and phalloidin staining

Morphological characters of the samples, gene expression patterns and F-actin dynamics were investigated as described previously using SEM [43], WMISH [27] and phalloidin staining [44], respectively. For all assays except SEM (WMISH, phalloidin staining and BrdU assay that is described below), at least 20 embryos were examined to ensure consistency between individuals. For SEM, since the embryos could not be rotated during the observation, we could only recorded a proportion of samples with the dorsal side facing up (typically five to ten in one trial); and we confirmed that they showed consistent morphological characters in the shell field.

#### BrdU assay

BrdU assay was performed in 6-well plates at 25 °C. BrdU (BBI Life Sciences, China; Cat. No. E607203) was dissolved in DMSO to prepare a 25-mM storage solution and stored at − 20 °C. Before use, the BrdU storage solution was diluted into a 1 mM solution using DMSO. At 6 or 7 hpf, BrdU was added to FSW to a final concentration of 1 μM. In control groups, the same volumes of DMSO was added. After one or two hours, the samples were collected at 8 hpf, and the embryos of BrdU and control groups showed no detectable morphological changes at the end of the treatment. The embryos were washed with FSW, fixed in 4% PFA at room temperature for 30–60 min, transferred to PBST and stored at 4 °C. Before immunostaining, the samples were incubated in 1 M HCl for 30 min to denature DNA. After washing with PBST, the samples were incubated in the blocking buffer (2% BSA in PBST) for 2 h at RT. The primary (anti-BrdU mouse monoclonal antibody [Sigma-Aldrich], 1:200) and second (Alexa Fluor 488 goat anti-mouse antibody [PTC], 1:200) antibodies were applied to detect the incorporated BrdU in the nuclei.

#### Microscopy

The samples were mounted in 90–100% glycerol and observed using a Nikon 80i microscope or a ZEISS LSM 710 laser-scanning confocal microscopy system.

## Supplementary information

**Additional file 1: Figure S1.** Expression of pSF genes in early embryos. The anterior/animal pole is on the top for each panel. The dorsal and ventral sides are difficult to discriminate at 3 and 4 hpf. Posttrocal expression of the genes (arrows) started at 5 hpf for BMP2/4, GATA2/3 and Hox1, while the earliest Engrailed expression was detected in dorsal cells at 4 hpf (k, which was used to determine the dorsal and ventral sides of the embryo). Pretrochal BMP2/4 expression was constantly detected at the stages investigated (arrowheads in a-c).

**Additional file 2: Figure S2.** No evident cell proliferation in the shell field from 6 to 8 hpf. Similar to Fig. 4, all panels were optical sections collected by an laser confocal microscope (note that the dorsal and ventral sides are different from those in Fig. 4). BrdU was added at 6 hpf and the samples were collected at 8 hpf. The incorporation of BrdU (green fluorescence) indicates the divided cells during this period. No evident cell proliferations was detected in the shell field (sf). D, dorsal; V, ventral. Bar represents 20 μm.

## Data Availability

The datasets analysed during the current study are available from the corresponding author on reasonable request.
