# Supplementary material for: Early shell field morphogenesis of a patellogastropod mollusk predominantly relies on cell movement and F-actin dynamics
Source: BMC Dev Biol. 2020 Aug 19;20:18. doi: 10.1186/s12861-020-00223-3 (PMC7439683; doi:10.1186/s12861-020-00223-3)
Supplement: Supplementary file 1 — Additional file 1: Figure S1. Expression of pSF genes in early embryos. The anterior/animal pole is on the top for each panel. The dorsal and ventral sides are difficult to discriminate at 3 and 4 hpf. Posttrocal expression of the genes (arrows) started at 5 hpf for BMP2/4, GATA2/3 and Hox1, while the earliest Engrailed expression was detected in dorsal cells at 4 hpf (k, which was used to determine the dorsal and ventral sides of the embryo). Pretrochal BMP2/4 expression was constantly detected at the stages investigated (arrowheads in a-c). [file 12861_2020_223_MOESM1_ESM.docx]

**Fig. S1**. Expression of pSF genes in early embryos. The anterior/animal pole is on the top for each panel. The dorsal and ventral sides are difficult to discriminate at 3 and 4 hpf. Posttrocal expression of the genes (arrows) started at 5 hpf for BMP2/4, GATA2/3 and Hox1, while the earliest Engrailed expression was detected in dorsal cells at 4 hpf (**k**, which was used to determine the dorsal and ventral sides of the embryo). Pretrochal BMP2/4 expression was constantly detected at the stages investigated (arrowheads in **a-c**).
