# Supplementary material for: Early shell field morphogenesis of a patellogastropod mollusk predominantly relies on cell movement and F-actin dynamics
Source: BMC Dev Biol. 2020 Aug 19;20:18. doi: 10.1186/s12861-020-00223-3 (PMC7439683; doi:10.1186/s12861-020-00223-3)
Supplement: Supplementary file 2 — Additional file 2: Figure S2. No evident cell proliferation in the shell field from 6 to 8 hpf. Similar to Fig. 4, all panels were optical sections collected by an laser confocal microscope (note that the dorsal and ventral sides are different from those in Fig. 4). BrdU was added at 6 hpf and the samples were collected at 8 hpf. The incorporation of BrdU (green fluorescence) indicates the divided cells during this period. No evident cell proliferations was detected in the shell field (sf). D, dorsal; V, ventral. Bar represents 20 μm. [file 12861_2020_223_MOESM2_ESM.docx]

**Fig. S2.** No evident cell proliferation in the shell field from 6 to 8 hpf. Similar to Fig. 4, all panels were optical sections collected by an laser confocal microscope (note that the dorsal and ventral sides are different from those in Fig. 4). BrdU was added at 6 hpf and the samples were collected at 8 hpf. The incorporation of BrdU (green fluorescence) indicates the divided cells during this period. No evident cell proliferations was detected in the shell field (sf). D, dorsal; V, ventral. Bar represents 20 μm.
